# Supplementary material for: Diversity and Anti-Infectious Components of Cultivable Rhizosphere Fungi Derived from Three Species of Astragalus Plants in Northwestern Yunnan, China
Source: J Fungi (Basel). 2024 Oct 24;10(11):736. doi: 10.3390/jof10110736 (PMC11595489; doi:10.3390/jof10110736)
Supplement: Supplementary file 1 [file jof-10-00736-s001.zip › jof-3184873-supplementary.pdf]

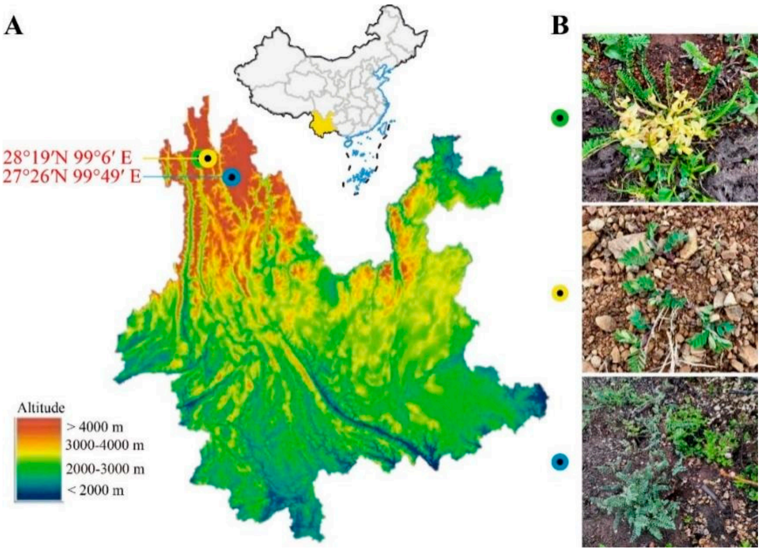

Figure S1 Map of sampling sites in the northwestern area of Yunnan province, China (A). In the picture B, the green dot is the sampling site for *Astragalus acaulis* (Aa) growing in high altitude grassland at 4,353 m; as shown in the yellow dot, *A. ernestii* (Ae) grows in the steep slopes of flowstone beaches at 4,048 m; the blue dot shows the *A. forrestii* (Af) survival environment, surrounded by more pine trees and deciduous foliage at 3,225 m [1].

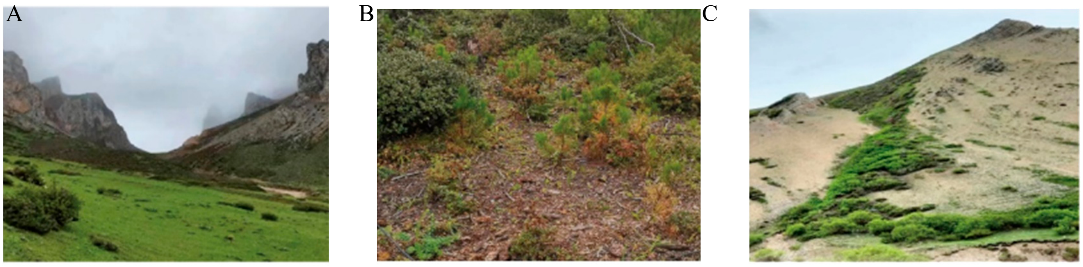

Figure S2 The plants of *Astragalus acaulis* (Aa) grow in alpine grassland (A), *A. forrestii* (Af) is surrounded by more pine trees and deciduous foliage (B), and *A. ernestii* (Ae) grows in the steep slopes of flowstone beaches (C) [1].

Table S1. Soil physicochemical properties of *Astragalus* acaulis (Aa), *A forrestii* (Af), and *A. ernestii* (Ae) [1].

Sample ID	PH	TN(g/kg)	TP(g/kg)	TK(g/kg)	SOM(g/kg)
Aa	5.60±0.01a	2.65±0.02a	1.66±0.02	18.33±0.10a	46.32±0.03a
Af	5.29±0.01a	3.37±0.02a	1.00±0.01	13.04±0.09a	55.12±0.79a
Ae	5.88±0.02a	2.18±0.02a	0.93±0.04	16.98±0.06a	40.12±0.13a

SOM, soil organic matter; TN, total nitrogen; TP, total phosphorus; TK, total potassium. Aa, *Astragalus acaulis*; Af, *Astragalus forrestii*; Ae, *Astragalus ernestii*. Data shown represent the average of three replicates and their standard deviations. The lowercase letter "a" indicates significant differences ($p < 0.05$) based on the Kruskal–Wallis test.

Table S2 A total of 7, 2 and 4 unique genera identified in the rhizosphere fungal community of *Astragalus acaulis* (Aa), *A. forrestii* (Af) and *A. ernestii* (Ae), respectively.

the name of fungal genera of Aa rhizosphere	the name of fungal genera of Af rhizosphere	the name of fungal genera of Ae rhizosphere
Truncatella	Lecanicillium	Leptosphaeria
Cordyceps	Umbelopsis	Fusarium
Exophiala		Nemania,
Beauveria		Aporospora
Samsoniella		
Neonectria		
Pseudogymnoascus		

Table S3 A total of 10, 15 and 7 unique species identified in the rhizosphere fungal community of *Astragalus acaulis* (Aa), *A. forrestii* (Af) and *A. ernestii* (Ae), respectively.

the name of fungal species of Aa rhizosphere	the name of fungal species of Af rhizosphere	the name of fungal species of Ae rhizosphere
Penicillium wellingtonense	Penicillium vasconiae	Penicillium brevicompactum
Aspergillus calidoustus	P. suaveolens	Aspergillus niger
Mortierella clonocystis	P. thomii	Trichoderma longibrachiatum
Truncatella angustata	Aspergillus versicolor	Leptosphaeria sclerotioides
Cordyceps farinosa	A. tabacinus	Fusarium solani
Exophiala tremulae	Mortierella minutissima	Nemania diffusa
Beauveria pseudobassiana	M. verticillata	Aporospora terricola
Samsoniella hepiali	Trichoderma paraviridescens	
Neonectria radicicola	T. koningiopsis	

Pseudogymnoascus roseus	T. viride
	T. longipile
	Lecanicillium aphanocladii
	Umbelopsis vinacea
	U. ramanniana
	U. nana

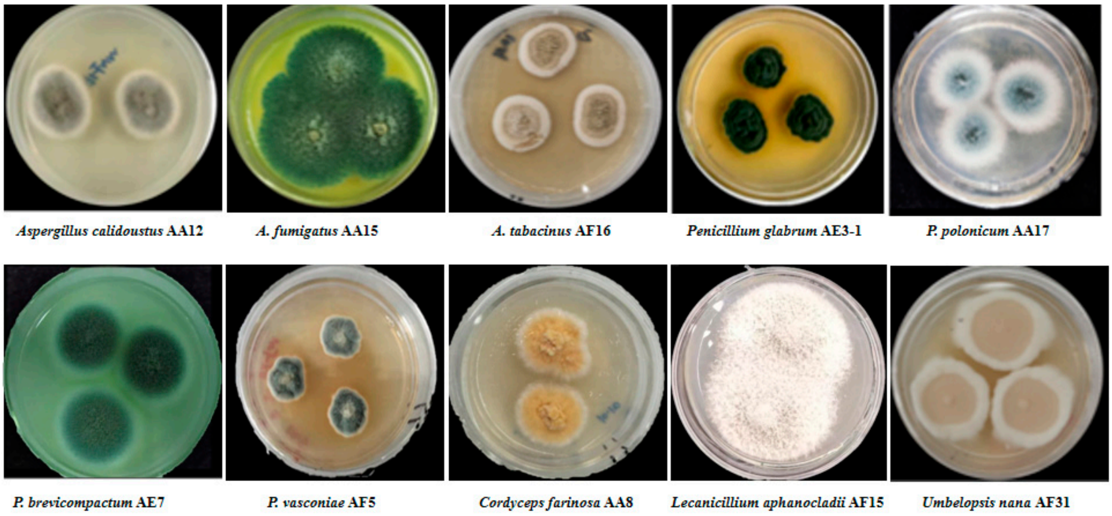

Figure S3 The micro-morphological characteristics of the colonies of the ten anti-infective fungal isolates.

A)

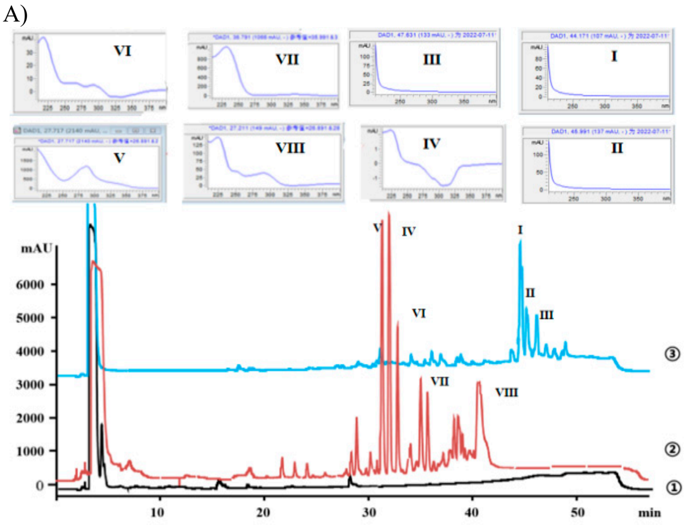

B)

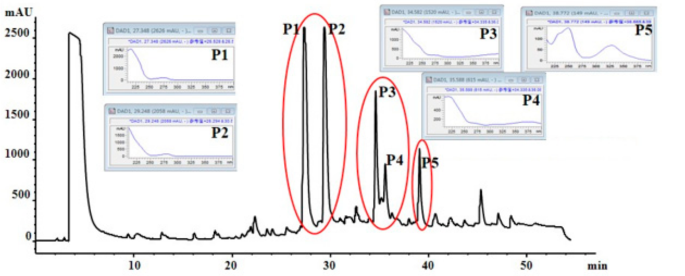
mAU
2500
2000
1500
1000
500
0
10
20
30
40
50
min
P1
P2
P3
P4
P5

C)

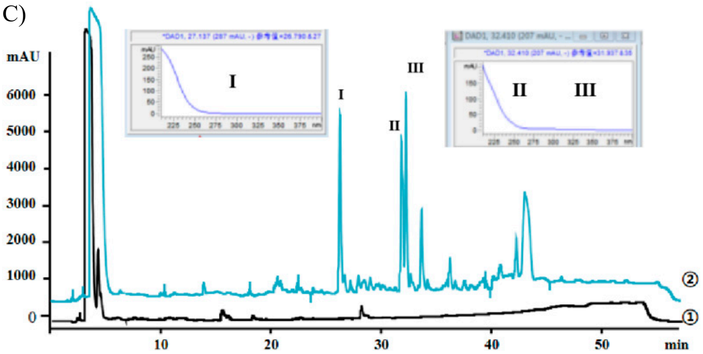
mAU
6000
5000
4000
3000
2000
1000
0
10
20
30
40
50
min
I
II
III
②
①

D)

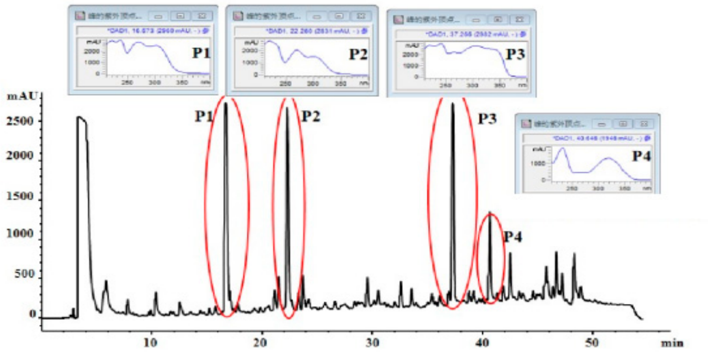
mAU
2500
2000
1500
1000
500
0
10
20
30
40
50
min
P1
P2
P3
P4

E)

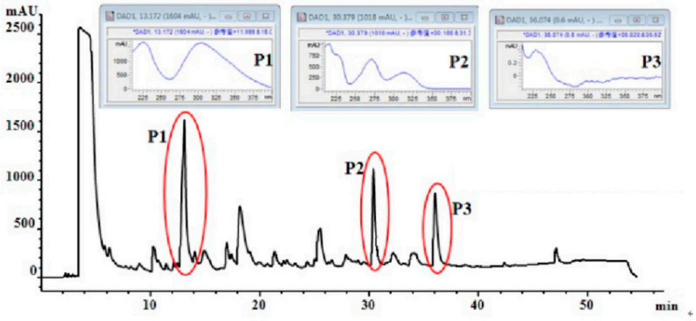
mAU
2500
2000
1500
1000
500
0
10
20
30
40
50
min
P1
P2
P3

F)

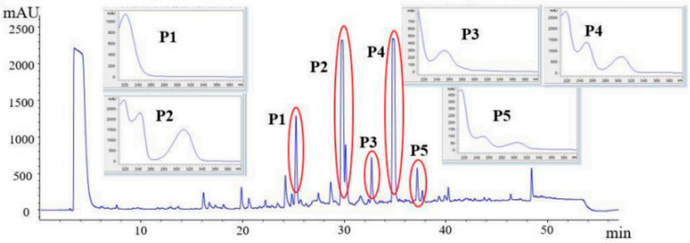
mAU
2500
2000
1500
1000
500
0
10
20
30
40
50
min
P1
P2
P3
P4
P5

G)

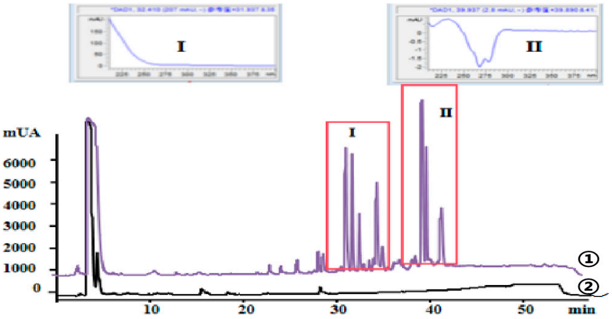

H)

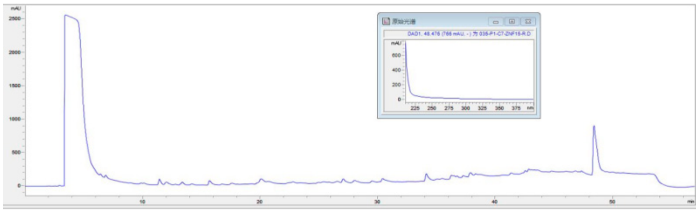

I)

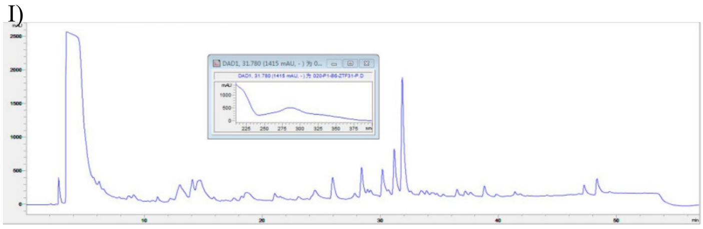

Figure S4 HPLC spectra of the crude ethyl acetate (EA) extracts of fermented substrates of *Aspergillus calidoustus* AA12 (A③) and the other nine target fungal strains of *A. fumigatus* AA15 (A②), *A. tabacinus* AF16 (B), *Penicillium polonicum* AA17 (C(②), *Penicillium vasconiae* AF5 (D), *P. glabrum* AF3-1 (E), *P. brevicompactum* AE7 (F), *Cordyceps farinosa* AA8 (G), *Lecanicillium aphanocladii* AF15 (H), and *Umbelopsis nana* AF31 (I).

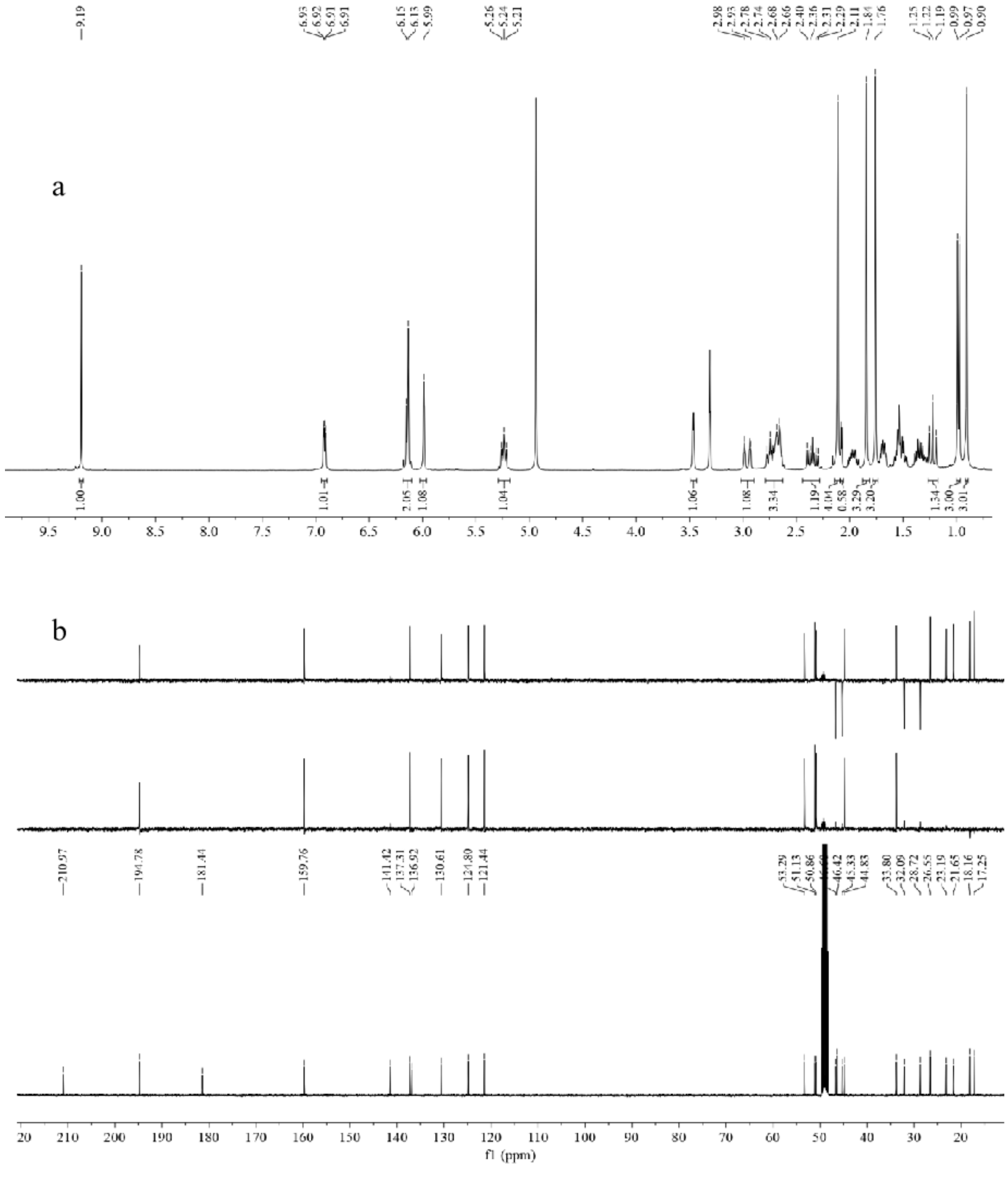

Figure S5 ^{1}H (a) and ^{13}C (b) NMR spectrum of 6-epi-ophiobolin G (**1**).

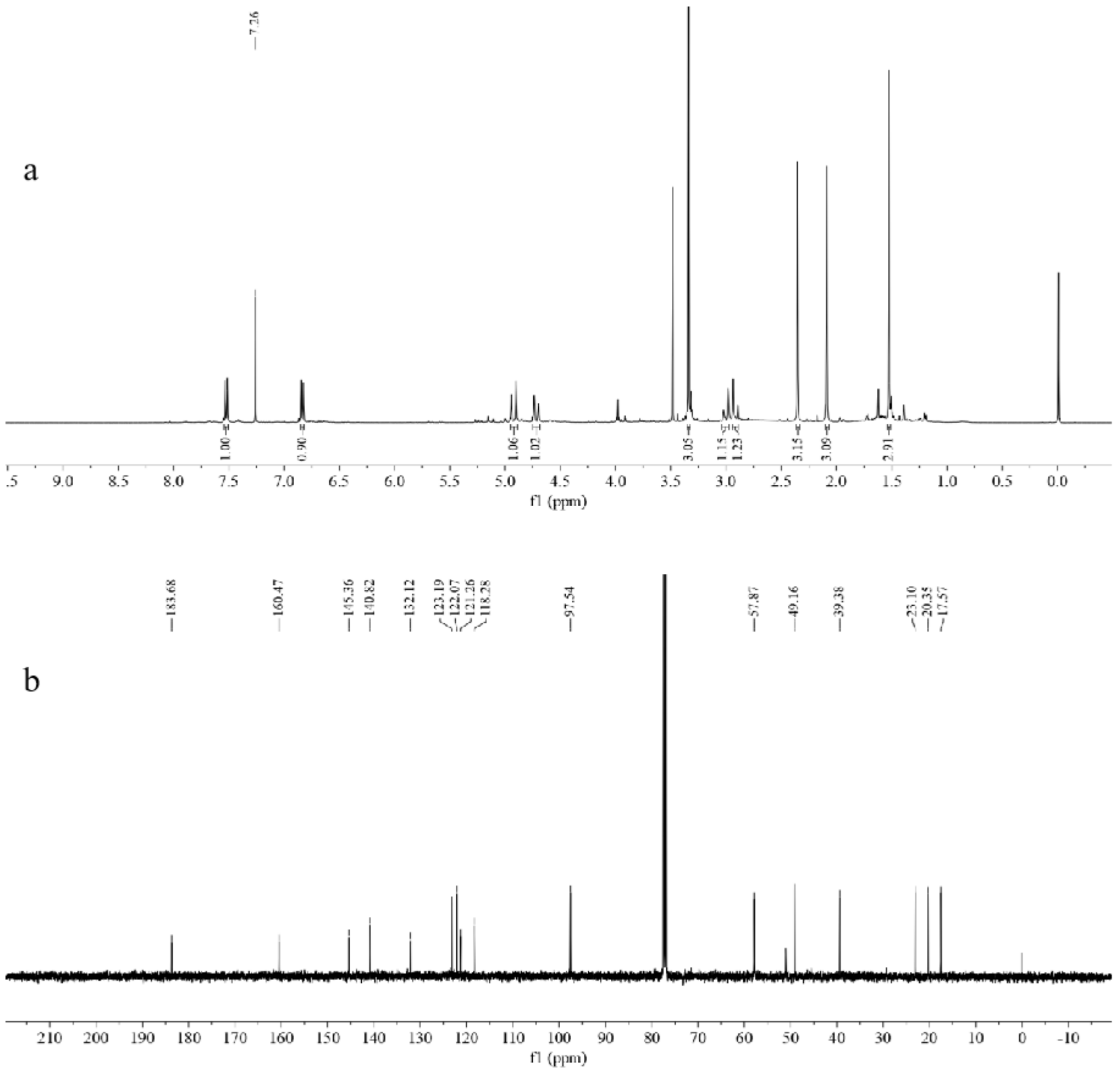

Figure S6 ^{1}H (a) and ^{13}C (b) NMR spectrum of penicisochroman A (**2**).

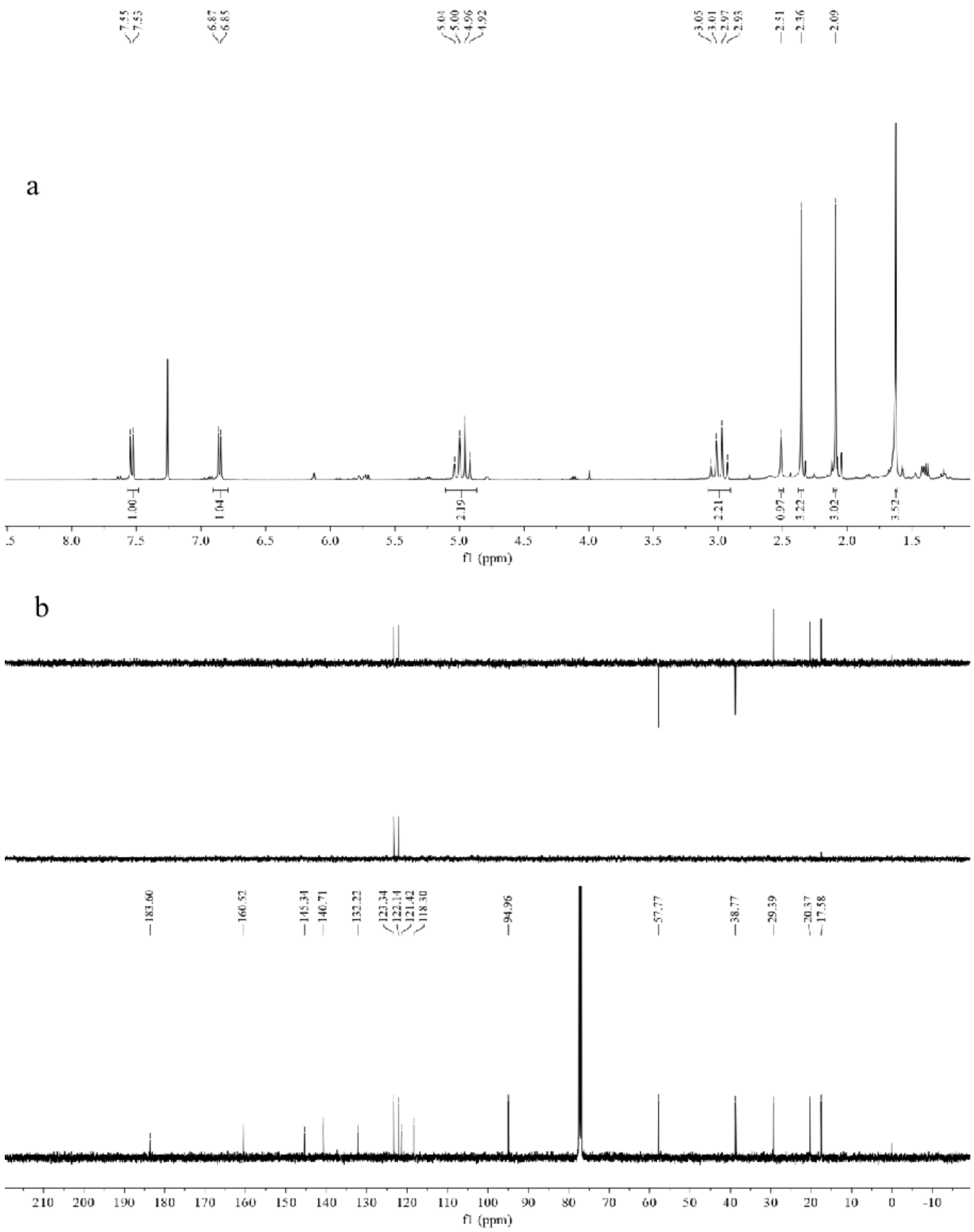

Figure S7 ^{1}H (a) and ^{13}C (b) NMR spectrum of pergillin (**3**).

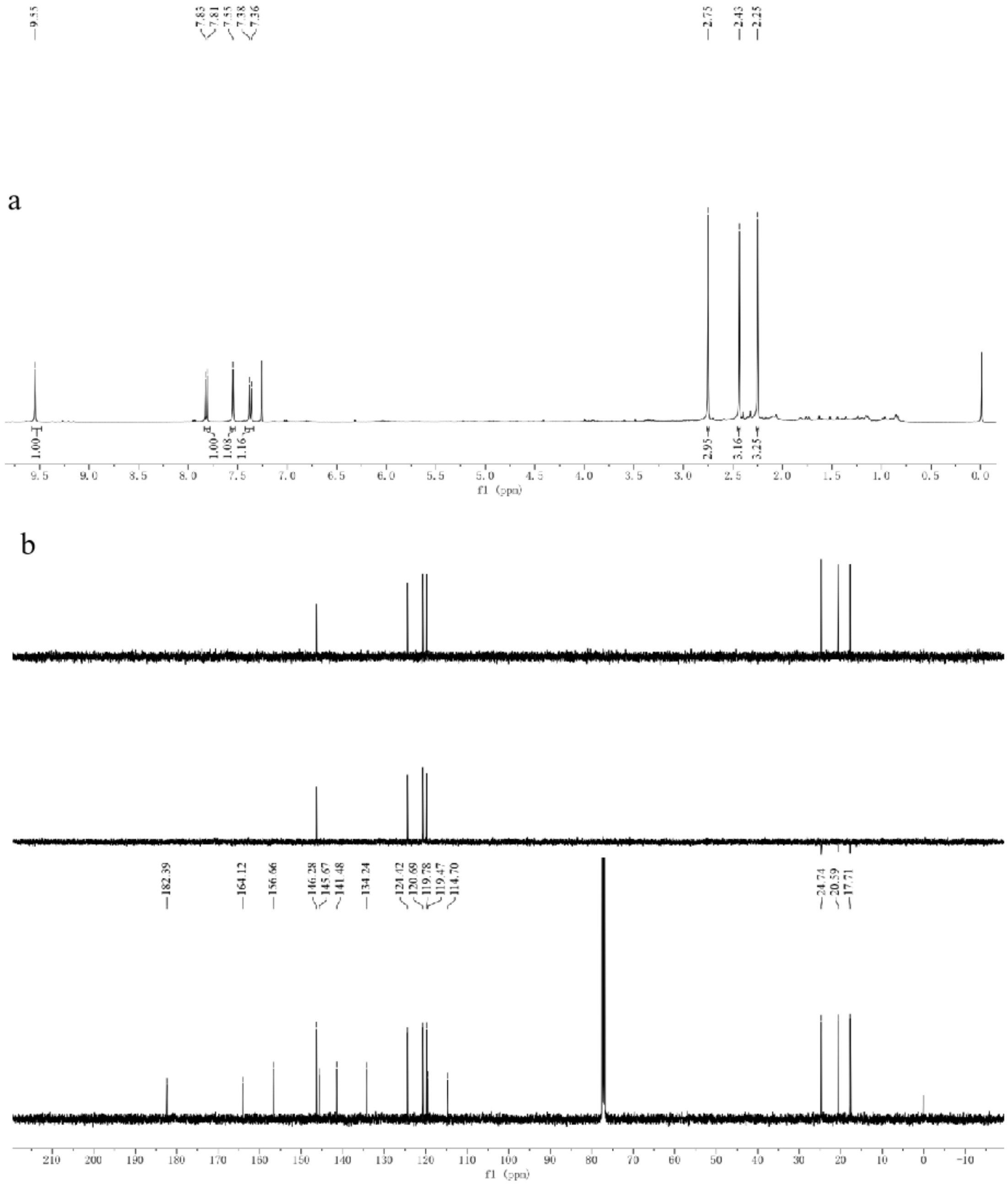

Figure S8 ^{1}H (a) and ^{13}C (b) NMR spectrum of 7-methyl-2-(1-methylethylethlidene)-furo[3,2-H]isoquinoline-3-one (**4**).

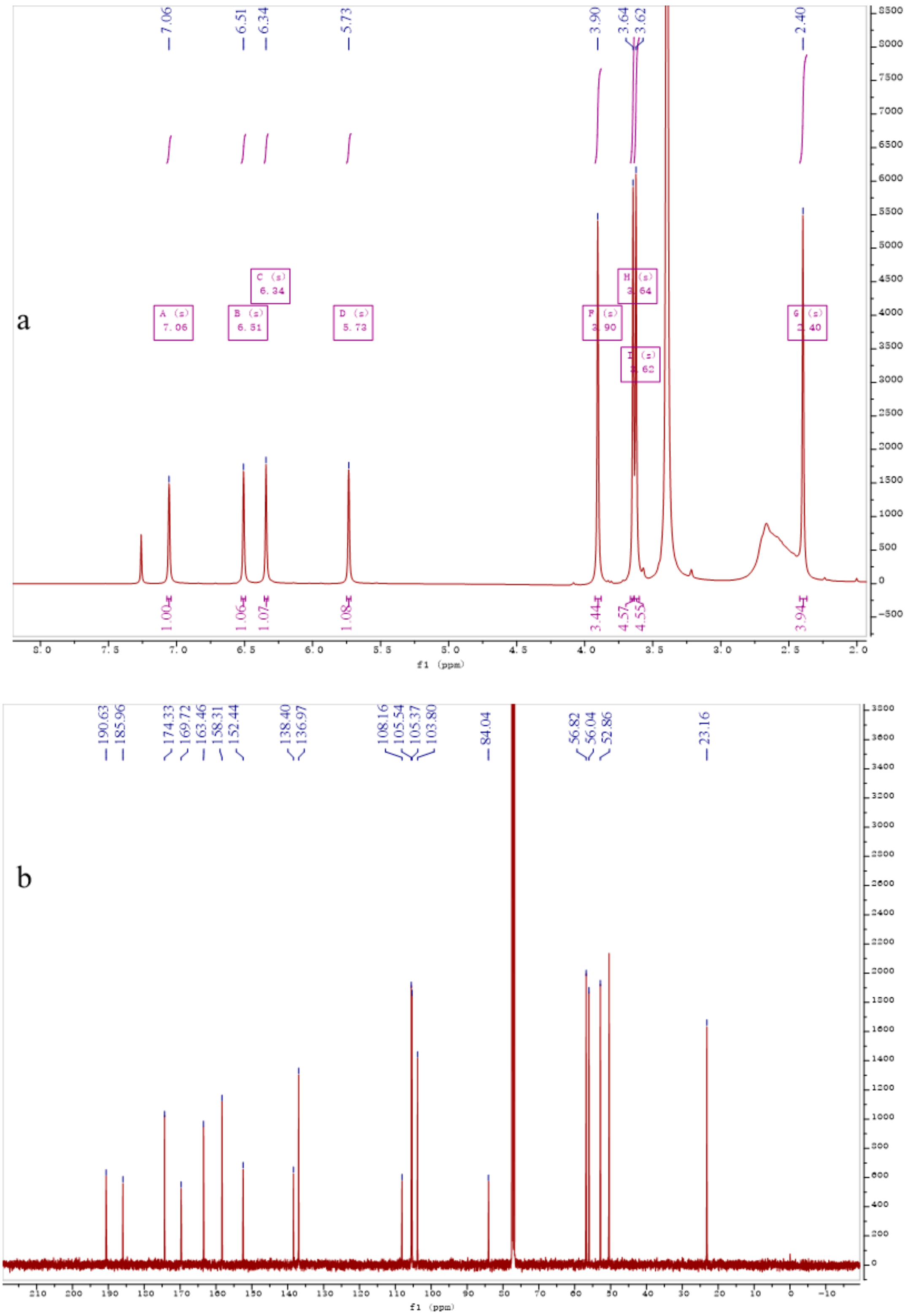

Figure S9 ^{1}H (a) and ^{13}C (b) NMR spectrum of trypacidin (**5**).

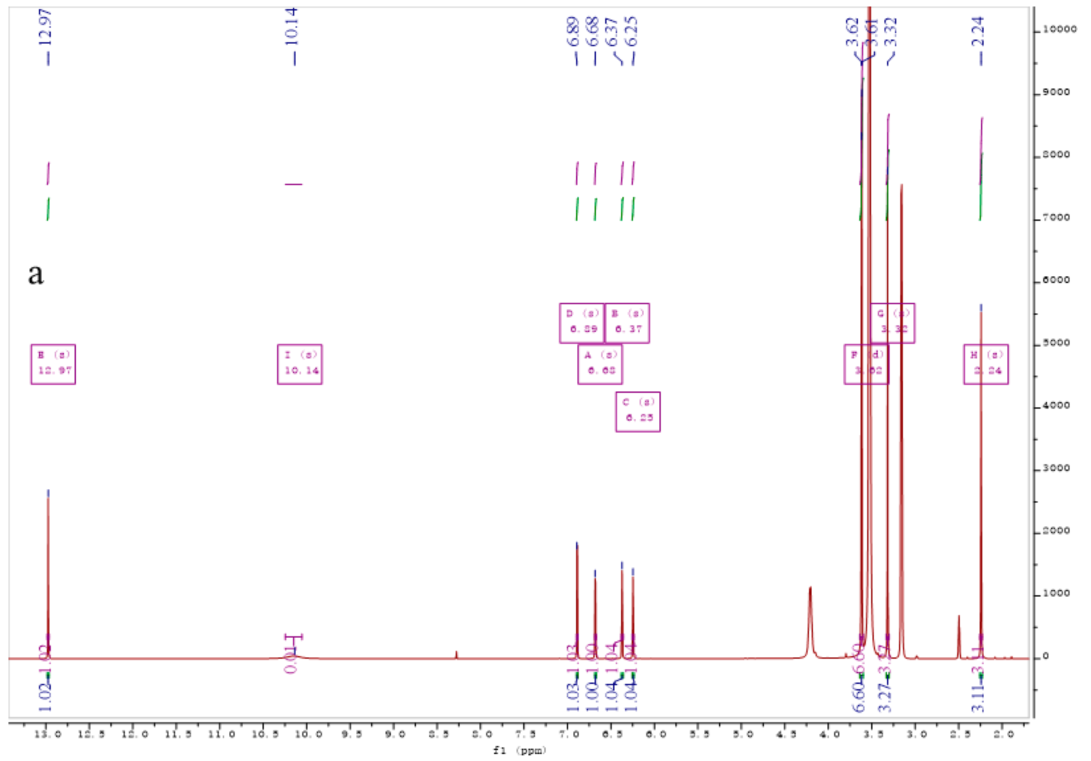

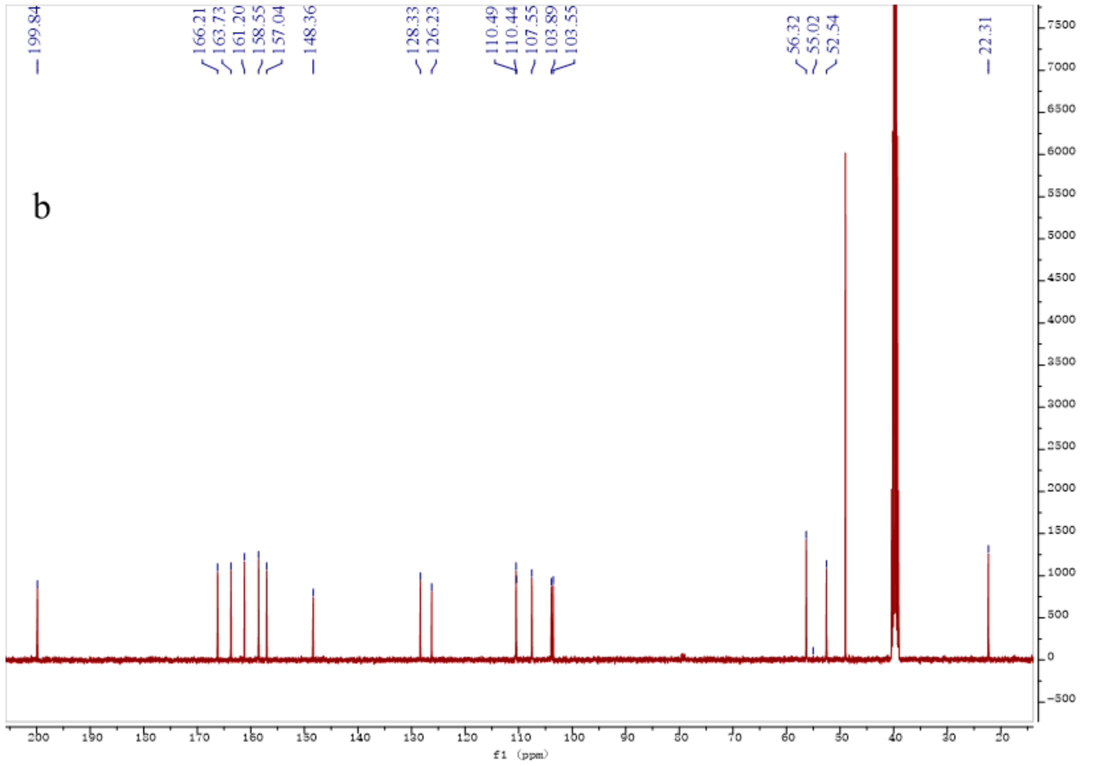

Figure S10 ^{1}H (a) and ^{13}C (b) NMR spectrum of 1,2-seco-trypacidin (**6**).

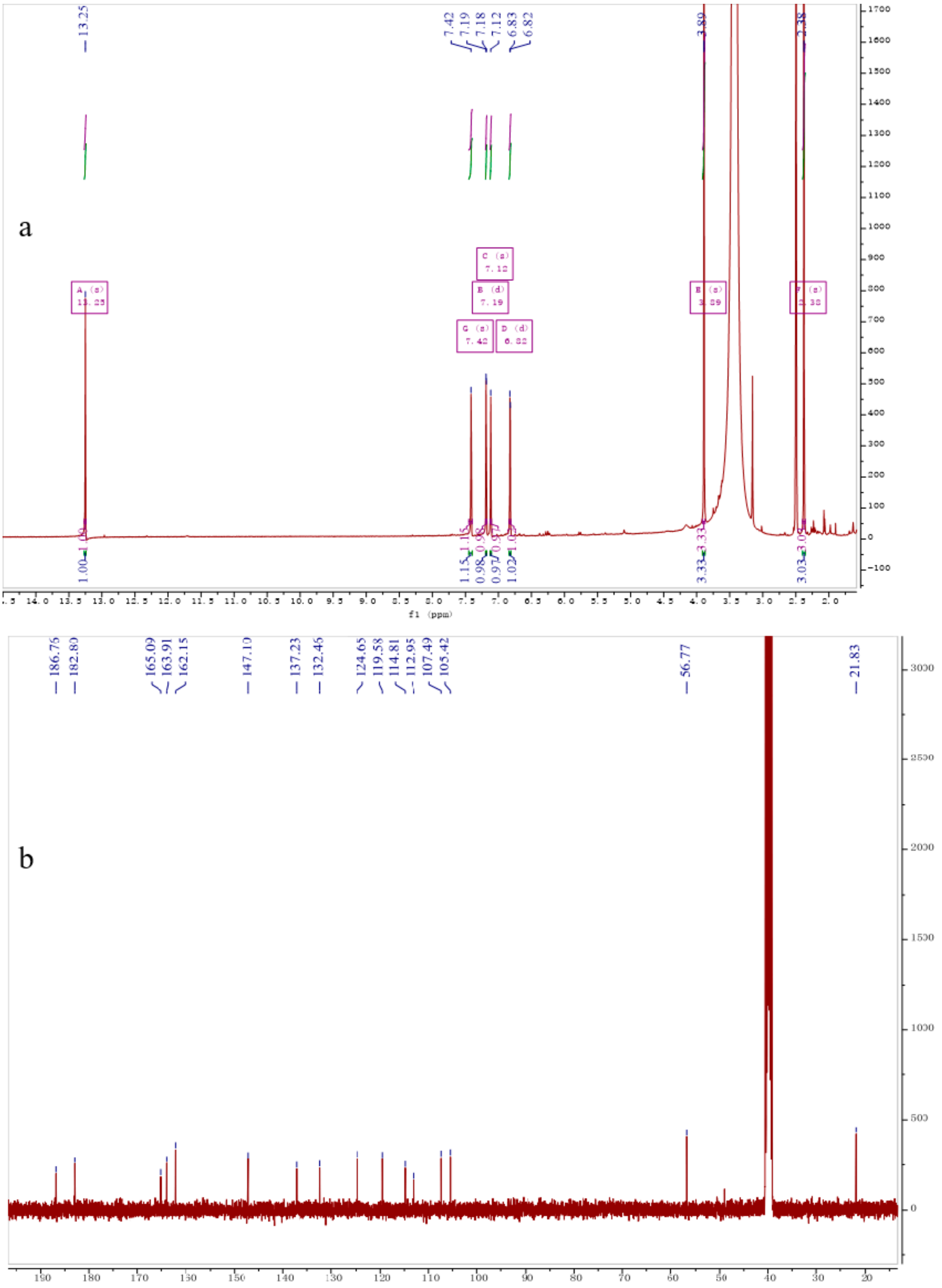

Figure S11 ^{1}H (a) and ^{13}C (b) NMR spectrum of questin (**7**).

Table S4 ^{1}H and ^{13}C NMR data of compounds **1** and **7** (δ in ppm).

No.	**1** (In CD_3OD)		**7** (In DMSO-d6)	
	$\delta_H{}^a$	$\delta_C{}^b$	$\delta_H{}^a$	$\delta_C{}^b$
1	1.19 t	45.33 CH_2		162.15 C
2	2.66 m	50.86 CH_2	7.12 s	124.65 CH
3		181.44 C		147.10 C
3-Me			2.38 s	21.83 CH_3
4	5.99 s	130.61 CH	7.42 s	119.58 CH
5		210.97 C	7.19 s	107.49 CH
6	3.46 d	51.13 CH		165.09 C
7		141.42 C	6.82 s	105.42 CH
8	6.91 d	159.76 CH		163.91 C
8-OMe			3.89 s	56.77 CH_3
9	2.93 d	32.09 CH_2		186.76 C
10	2.61 m	44.83 CH		182.80 C
11		46.68 C		137.23 C
12	1.49 m; 1.52 m	46.42 CH_2		112.95 C
13	1.28 m	28.72 CH_2		114.81 C
14	1.87 m	50.29 CH		132.46 C
15	2.68 m	33.80 CH		
16	5.21 t	137.31 CH		
17	6.13 t	124.80 CH		
18	5.99 t	121.44 CH		

19		136.92 C
20	2.11 s	17.20 CH_3
21	9.19 s	194.78 CH
22	0.90 s	23.19 CH_3
23	0.97 d	21.65 CH_3
24	1.76 s	18.16 CH_3
25	1.84 s	26.55 CH_3

a Recorded at 400 MHz; b Recorded at 100 MHz.

Table S5 ^{1}H and ^{13}C NMR data of compounds **2–4** (δ in ppm).

No.	**2** (In $CDCl_3$)		**3** (In $CDCl_3$)		**4** (In $CDCl_3$)	
	δ_H[a]	δ_C[b]	δ_H[a]	δ_C[b]	δ_H[a]	δ_C[b]
2		145.34 C		145.34 C		145.67 C
3		183.59 C		183.40 C		182.39 C
3a		121.28 C		121.42 C		119.47 C
4	7.53 d	122.02 CH	7.53 d	122.14 CH	7.78 d	124.42 CH
5	6.85 d	123.16 CH	6.87 d	123.34 CH	7.36 d	119.78 CH

5a		140.77 C		140.71 C		141.48 C
6	3.00 dd	39.38 CH_2	2.93 dd	38.77 CH_2	7.55 s	120.69 CH
7		97.50 C		94.96 C		156.66 C
9	4.93 dd	57.86 CH_2	4.92 dd	57.77 CH_2	9.51 s	146.28 CH
9a		118.29 C		118.30 C		114.70 C
9b		160.45 C		160.52 C		164.12 C
10	1.53 s	23.10 CH_3	1.63 s	29.39 CH_3	2.75 s	24.74 CH_3
11		131.85 C		132.22 C		134.22 C
12	2.09 s	20.31 CH_3	2.09 s	17.58 CH_3	2.43 s	17.71 CH_3
13	2.36 s	17.53 CH_3	2.36 s	20.37 CH_3	2.55 s	20.59 CH_3
7-OMe	3.55 s	49.13 CH_3				

a Recorded at 400 MHz; b Recorded at 100 MHz.

Table S6 ^{1}H and ^{13}C NMR data NMR data of compounds **5** and **6** (δ in ppm).

No.	**5** (In $CDCl_3$)		**6** (In DMSO-d6)	
	$\delta_H{}^a$	$\delta_C{}^b$	$\delta_H{}^a$	$\delta_C{}^b$
2		84.04 C		126.23 C
3		190.63 C		199.84 C
3a		108.16 C		110.44 C
4		158.31 C		161.20 C
5	6.34 s	105.37 CH	6.37 s	110.49 CH
6		152.44 C		148.36 C
6a	2.40 s	23.16 CH_3	2.24 s	22.31 CH_3
7	6.51 s	105.54 CH	6.68 s	103.89 CH

7a		174.33 C		163.73 C
1′		138.40 C		128.33 C
2′	7.06 s	136.97 CH	6.89 s	107.55 CH
3′		185.96 C		158.55 C
4′	5.73 s	103.80 CH	6.25 s	103.55 CH
5′		169.72 C		157.04C
1′-COOMe		163.46 C		166.21 C
4-OMe	3.90 s	56.04 CH_3	3.32 s	52.54CH_3
5′-OMe	3.62 s	56.82 CH_3	3.62 s	56.32CH_3
1′-COOMe	3.64 s	52.86 CH_3	3.61 s	55.02 CH_3

a Recorded at 400 MHz; b Recorded at 100 MHz.

References

1. Ding, J.J.; Zhou, G.J.; Chen, X.J.; Xu, W.; Gao, X.M.; Zhang, Y.Z.; Jiang, B.; Li, H.F.; Wang, K.L. Analysis of microbial diversity and community structure of rhizosphere soil of three *Astragalus* species grown in special high-cold environment of northwestern Yunnan, China. Microorganisms **2024**, 12, 539.
